# Primary microcephaly gene CENPE is a novel biomarker and potential therapeutic target for non-WNT/non-SHH medulloblastoma

**DOI:** 10.3389/fimmu.2023.1227143

**Published:** 2023-08-01

**Authors:** Huangyi Fang, Yusong Zhang, Chengyin Lin, Zhenkai Sun, Wei Wen, Hansong Sheng, Jian Lin

**Affiliations:** ^1^ Wenzhou Medical University, Wenzhou, China; ^2^ Department of Neurosurgery, The Second Affiliated Hospital of Wenzhou Medical University, Wenzhou, China; ^3^ Department of Surgery, The First People’s Hospital of Jiashan, Jiaxing, China; ^4^ The Key Laboratory of Pediatric Hematology and Oncology Diseases of Wenzhou, The Second Affiliated Hospital of Wenzhou Medical University, Wenzhou, China

**Keywords:** CENPE, non-WNT/non-SHH medulloblastoma, microcephaly, bioinformatics analysis, cell cycle, p53

## Abstract

**Background:**

Non-WNT/non-SHH medulloblastoma (MB) is one of the subtypes with the highest genetic heterogeneity in MB, and its current treatment strategies have unsatisfactory results and significant side effects. As a member of the centromere protein (CENP) family, centromeric protein E (CENPE) is a microtubule plus-end-directed kinetochore protein. Heterozygous mutations in CENPE can leads to primary microcephaly syndrome. It has been reported that CENPE is upregulated in MB, but its role in MB development is still unknown.

**Methods:**

We downloaded the relevant RNA seq data and matched clinical information from the GEO database. Bioinformatics analysis includes differential gene expression analysis, Kaplan-Meier survival analysis, nomogram analysis, ROC curve analysis, immune cell infiltration analysis, and gene function enrichment analysis. Moreover, the effects of CENPE expression on cell proliferation, cell cycle, and p53 signaling pathway of non-WNT/non-SHH MB were validated using CENPE specific siRNA *in vitro* experiments.

**Results:**

Compared with normal tissues, CENPE was highly expressed in MB tissues and served as an independent prognostic factor for survival in non-WNT/non-SHH MB patients. The nomogram analysis and ROC curve further confirmed these findings. At the same time, immune cell infiltration analysis showed that CENPE may participate in the immune response and tumor microenvironment (TME) of non-WNT/non-SHH MB. In addition, gene enrichment analysis showed that CENPE was closely related to the cell cycle and p53 pathway in non-WNT/non-SHH MB. *In vitro* experimental validation showed that knockdown of CENPE inhibited cell proliferation by activating the p53 signaling pathway and blocking the cell cycle.

**Conclusion:**

The expression of CENPE in non-WNT/non-SHH MB was positively correlated with poor prognosis. CENPE may affect tumor progression by regulating cell cycle, p53 pathway, and immune infiltration. Hence, CENPE is highly likely a novel biomarker and potential therapeutic target for non-WNT/non-SHH MB.

## Introduction

Medulloblastoma (MB) is the most common cerebellar malignant tumor in children, which accounts for a large proportion of both cancer-related incidence rate and mortality in this age group (1). MB is divided into at least four subgroups: Wingless-activated (WNT), Sonic Hedgehog-activated (SHH), Group 3 and Group 4, which are based on different molecular expression profiles, clinical manifestations, and prognostic characteristics (2). In 2021, the Groups 3 and Group 4 of MB were summarized as non-WNT/non-SHH subtype in the WHO classification of central nervous system tumors (3). The characteristic of this subtype is high heterogeneity, with varying biological characteristics, genetic basis, and clinical course. This subtype accounts for approximately 60% of all cases and remains the most genetically heterogeneous and least understood subset of MB cases (4). In terms of treatment, there is still a lack of effective treatment targets for non-WNT/non-SHH MB. The current treatment methods mainly include surgical resection combined with postoperative cranial spinal cord irradiation (CSI) and chemotherapy. Although the survival period of some children has been extended, their mortality rate is still high, and some children cannot tolerate the side effects of radiation and chemotherapy, resulting in poor long-term quality of life and frequent neurological and endocrine sequelae (5). Therefore, it is necessary to search for new biomarkers and safer and more effective therapeutic targets for non-WNT/non-SHH MB.

As a member of the centromere protein (CENP) family, centromeric protein E (CENPE) is a microtubule plus-end-directed kinetochore protein. It plays an important role in spindle assembly checkpoint (SAC), chromosome congression and spindle microtubule capture at kinetochores during mitosis (6, 7). Pan-cancer analysis indicates that CENPE is associated with various types of tumorigenesis. Its high expression is associated with poor prognosis of non-small cell lung cancer, colorectal cancer and breast cancer (8–10). In addition, CENPE is involved in the malignant proliferation and migration of lung adenocarcinoma and ovarian cancer (11, 12).

In research on the nervous system, it has been found that that as a key gene in primary microcephaly (MCPH) syndrome, CENPE mutations can lead to a decrease in brain capacity (13). However, inhibiting CENPE expression in MB cells can cause p53 dependent cell apoptosis (14). Although the origin of MB is unknown, it is clear that MB cells share many common molecular features with radial glial cells and cerebellar granulosa progenitor cells. Based on the above foundation, mutated genes in primary MCPH syndrome have been proposed as potential therapeutic targets for MB (15).

In this study, we evaluated the expression changes of CENPE in MB and its potential value in diagnosis and prognosis, and explored the relationship between CENPE and immune infiltration. In addition, enrichment analysis showed that CENPE is associated with cell cycle, p53 signaling pathway in non-WNT/non-SHH MB, and this result was validated in cell experiments. In summary, our results indicate that CENPE can serve as a novel biomarker and potential therapeutic target for non-WNT/non-SHH MB.

## Materials and methods

### The expression levels of CENPE

GSE124814 and GSE85217 datasets were retrieved from the GEO database (https://www.ncbi.nlm.nih.gov/geo/). The platform for GSE124814 included the transcriptome sequencing data of 1350 MB samples and 291 normal cerebellum samples. The platform for GSE85217 included 763 MB samples of transcriptome sequencing data and matched clinical information. Box plot and scatter plot were used to compare the expression levels of CENPE in MB samples and normal cerebellum tissues.

### Survival analysis

The median expression of CENPE was selected to divided the patients into CENEP^high^ expression group and CENPE^low^ expression group. The R software package survminer was used to analyze the correlation between CENPE expression and the overall survival of patients with MB. The relationship between CENPE expression and prognosis of MB patients was analyzed by Spearman and logistic regression analysis, and visualized by R package ggplot2.

### Predictive nomogram construction

We used the R package rms to integrate data on age, gender, tumor subtypes, and survival time. Using Cox proportional hazard regression analysis established a nomogram to evaluate the prognostic significance of these features in 599 samples. We evaluated the diagnostic and predictive capabilities of CENPE by drawing Receiver operating characteristic (ROC) curves. The area under the curve (AUC) was calculated, AUC>0.7 indicates satisfactory predictive ability.

### Immune infiltration analysis

We selected the TIMER method in the R package IOBR to evaluate the infiltration level of immune cells in non-WNT/non-SHH MB sample in dataset GSE85217. Spearman correlation analysis was used to analyze the correlation between CENPE expression and the infiltration of immune cells, which was visualized by R package ggplot2 (4.1.3).

### Functional enrichment analysis

We analyzed the correlation between CENPE and all genes in the GSE85217 dataset through the R package tidyverse, and after ranking according to correlation, the 100 genes with the strongest positive correlation and the 100 genes with the strongest negative correlation with CENPE were selected. These 200 genes were subjected to Gene Ontology (GO) and Kyoto encyclopedia of genes and genomes (KEGG) analysis using the R package clusterProfiler. Gene set enrichment analysis (GSEA) was performed using the GSEA software (version 3.0) derived from GSEA (http://software.broadinstitute.org/gsea/index.jsp) to investigate the differences in biological functions and KEGG pathways between the CENEP^high^ and CENPE^low^ non-WNT/non-SHH MB groups (cutoff value was 50%). To evaluate relevant pathways and molecular mechanisms, based on gene expression profiling and phenotype grouping, with a minimum gene set of 5 and a maximum gene set of 5000, with 1000 resampling, *P* < 0.05 and FDR < 0.25 were considered statistically significant.

### Protein–protein interaction network analysis

The protein-protein interaction (PPI) network of CENPE co-expressed genes in non-WNT/non-SHH MB was analyzed using the search tool (STRING database, https://string-db.org/, version 11.0b) for the retrieval of interacting genes. The Betweenness score of each node analyzed by Cytoscape 3.7.2, and the top 50 with the highest score were screened to draw the skeleton network and correlation heat map.

### Cell culture and transfection

The non-WNT/non-SHH MB cell lines D283 and D341 were purchased from ATCC and cultured in Dulbecco’s modified Eagle’s medium (DMEM) (Gibco, Waltham, MA, USA) containing 10% fetal bovine serum (FBS) (Gibco, Waltham, MA, USA) at 37°C (16). The cells were grown to ~50% confluency in six-well plates before transfection. We transfected the small interfering RNA (siRNA) sequences with Lipofectamine 3000 (Invitrogen, San Diego, CA, USA) according to the manufacturer’s instructions. The siRNA sequences of CENPE in D283 and D341 cells were used as follows: siRNA-CENPE (5′-GGCUGUAAUAUAAAUCGAA-3′), and siRNA negative control (NC) (5′-TTCTCCGAACGTCACGT-3′).

### Western blot

D283 and D341 cells were lysed with protease inhibitor, and RIPA cleavage buffer (Thermo Fisher Scientific, Shanghai, China). The protein lysates were resolved *via* sodium dodecyl sulfate polyacrylamide gel electrophoresis (SDS-PAGE) and imprinted on polyvinylidene difluoride (PVDF) membranes (Millipore, Billerica, MA, USA) for analysis. Anti-CENEP (1:1,000 dilution, DF7745; Affinity Biosciences, OH, USA), anti-P53 (1:500 dilution, AF0879; Affinity Biosciences, OH, USA), anti-P21 (1:500 dilution, AF6290; Affinity Biosciences, OH, USA), anti-CDK1 (1:500 dilution, DF6024; Affinity Biosciences, OH, USA) and anti-β-Actin (1:5,000 dilution, AF7018; Affinity Biosciences, OH, USA) were incubated overnight. Goat Anti-Rabbit IgG (H+L) HRP-conjugated secondary antibodies (1:5,000 dilution; S0001, Affinity Biosciences, OH, USA) were added at room temperature for 2 h. The membrane was detected with BeyoECL Plus developer (Beyotime, Shanghai, China).

### Cell cycle analysis

D283 and D341 cells were cultured in six-well plates with 2 × 10^5^ cells for 24 h and were transfected with CENPE siRNA for 48 h. The cells were collected, and then fixed with 70% ethanol at 4°C overnight. The cells were stained with RNase A-containing PI buffer (C1052; Beyotime, Shanghai, China) at 37°C for 30 min. The cell cycle distribution was detected by flow cytometry (Beckman Coulter Quanta SC System).

### Cell counting kit-8 assay

D283 and D341 cells transfected with CENPE siRNA were cultured in a 96-well plate at 1 × 10^3^/well and allowed to adhere overnight. 10 μL CCK-8 solution was added to each well and incubated for 1.5 h. A microplate reader (Thermo Fisher Scientific, Shanghai, China) was used to detected the absorbance at 450 nm every day.

### Immunofluorescence staining

D283 and D341 cells transfected with CENPE siRNA were fixed with 4% paraformaldehyde for 15 min at room temperature, and permeabilized with 0.2% TritonX-100 for 15 min. After blocking in 10% goat serum for 30 min, the cells were incubated with anti-ki67 (1:200 dilution, AF0198; Affinity Biosciences, OH, USA) antibody at 4°C overnight. Then the cells were incubated with AlexaFluor 488 (goat anti-rabbit IgG, Abcam, Cambridge, UK, 1:1,000 dilution) at room temperature for 40 min. At last, the cells were stained with Dapi (1 μg/ml) for 10 min in dark place at room temperature and viewed with an inverted IX71 microscope system (Olympus, Tokyo, Japan). The mean intensity was measured by ImageJ software.

### Statistical analysis

R (4.1.3) software and GraphPad Prism (8.0.0) software were used for statistical analyses, the main R packages used in the study were as follows: “survminer”, “tidyverse”, “IOBR”, “clusterProfiler”, “rms” and “ggplot2”. All in experiments were repeated three times. A t-test was used in comparing the means of two groups, *p* < 0.05 means statistical significance.

## Results

### CENPE was highly expressed and served as an independent prognostic factor for overall survival in MB

**Figure 1:**
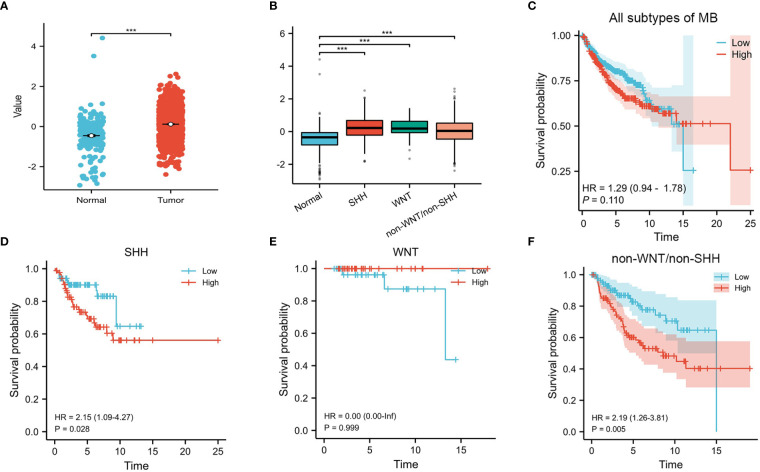
CENPE’s overall expression and survival analyses in MB. **(A, B)** The expression of CENPE in different subtypes of MB and normal cerebellum tissue. **(C–F)** Kaplan–Meier survival curve for impacts of CENPE on overall survival of all subtypes MB patients. ***p < 0.001.

Our results showed that the expression of CENPE was significantly higher in MB than in normal cerebellum tissue (**Figure 1A**). Moreover, the expression of CENPE was also elevated in different subtypes of MB (**Figure 1B**). Kaplan-Meier survival analysis showed that the expression of CENPE in MB was not statistically significant with the survival prognosis of MB patients (**Figure 1C**). However, the expression of CENPE in the non-WNT/non-SHH subtype and SHH subtype was negatively correlated with the survival prognosis of patients. The Hazard Ratio (HR) value of CENPE in survival of non-WNT/non-SHH MB patients was higher than that in SHH subtype (**Figures 1D–F**).

### Diagnostic and predictive ability of CENPE in MB

**Figure 2:**
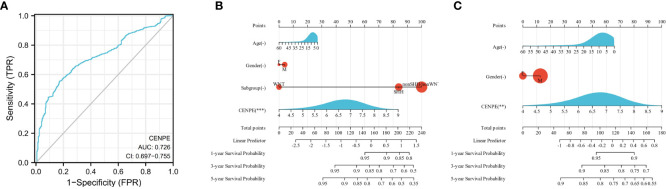
Predictive ability of CENPE for MB. **(A)** ROC curve analysis of CENPE expression in MB and normal cerebellum tissues. **(B)** Nomograms to predict 1 -, 3 -, and 5-year survival for patients with all subtypes MB. **(C)** Nomograms to predict 1 -, 3 -, and 5-year survival of patients with non-SHH/non-WNT MB. **p < 0.01 and ***p < 0.001.

To analyze the diagnostic value of CENPE expression in MB, we performed ROC curve and nomogram analysis on the CENPE expression data of GSE85217 database to evaluate the diagnostic value of the gene. The area under the ROC curve (AUC) was 0.726 (**Figure 2A**), indicating the good predictive ability. We combined the expression level of CENPE with clinical information to construct a nomogram to predict 1-, 3-, and 5-year survival in patients with MB. The nomogram showed that the subtypes of MB and the expression of CENPE were good prognostic indicators (**Figure 2B**). We further constructed a nomogram among the non-WNT/non-SHH subtypes with the worst prognosis in MB to predict 1-, 3-, and 5-year survival of patients. The results indicated that prognostic prediction of the expression level of CENPE in non-WNT/non-SHH MB was better than other traditional clinical features (**Figure 2C**). Moreover, the results of **Figures 1C–F** indicated that among all subtypes of MB patients, high expression of CENPE was most strongly correlated with poor prognosis in non-WNT/non-SHH MB. Therefore, we chose to further investigate the role of CENPE in the occurrence and development of non-WNT/non-SHH MB.

### The correlation between CENPE and immune cell infiltration in non-WNT/non-SHH MB

**Figure 3:**
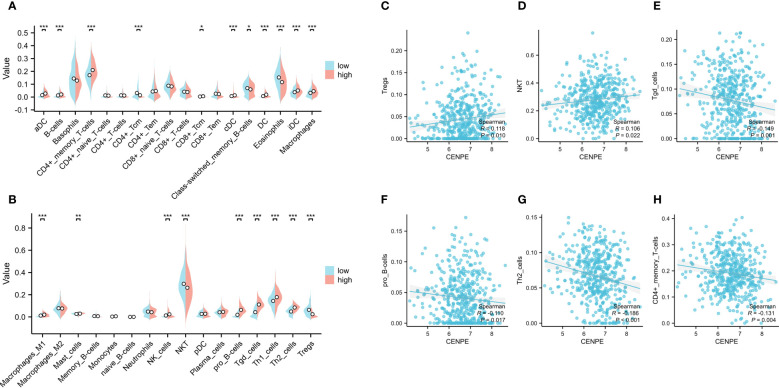
Correlation analysis between CENPE expression and immune infiltration in non-WNT/non-SHH MB. **(A, B)** Differential distribution of immune cells in patients with high and low CENPE expression. Correlation between CENPE expression and immune infiltration in non-WNT/non-SHH MB: **(C)** Tregs, **(D)** NKT, **(E)** Tgd cells, **(F)** pro B cells, **(G)** TH2 cells, **(H)** CD4+ Tem. *p < 0.05, **p < 0.01 and ***p < 0.001.

To explore the correlation between the expression level of CENPE and tumor immune response, we first used CIBRSORT to evaluate the differences in immune cell infiltration in non-WNT/non-SHH MB with high and low expression of CENPE. The results showed that CD4+ central memory T cells (Tcm), Class-switched memory B cells, Eosinophils, NKT, regulatory T cells (Tregs) were highly infiltrated in the low CENPE expression group. In contrast, the expression of Activated dendritic cells (aDC), B cells, CD4+ memory T cells (Tem), CD8+ Tcm, DC, conventional DC, immature DC, Macrophages, Macrophages M1, Mast cells, NK cells, pro B-cells, Tgd cells, Th1 cells, Th2 cells were highly infiltrated in the high CENPE expression group (**Figures 3A, B**). We further analyzed the correlation between the expression level of CENPE and immune infiltration in non-WNT/non-SHH MB. The results showed that CENPE expression correlated positively with the infiltration of Tergs (r=0.118, p=0.010), NKT(r=0.106, p=0.022), but negatively with Tgd cells (r=-0.149, p=0.001), pro B cells (r=-0.110, p=0.0017), Th2 cells (r=-0.186, p<0.001), CD4+ Tem (r=-0.131, p=0.004) (**Figures 3C–H**).

### Biological pathways correlated with CENPE in non-WNT/non-SHH MB

**Figure 4:**
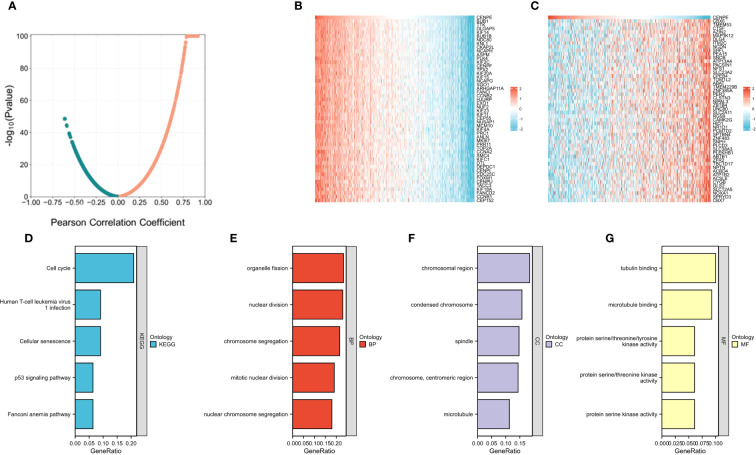
Functional Enrichment Analysis of CENPE co-expressed genes in non-WNT/non-SHH MB. **(A)** Volcano map showed CENPE co-expression genes in non-WNT/non-SHH MB. **(B, C)** Heat maps showed top 50 genes positively and negatively correlated to CENPE. **(D)** Enrichment analysis of KEGG terms for CENPE co-expression genes. **(E–G)** Enrichment analysis GO terms for CENPE co-expression genes.

**Figure 5:**
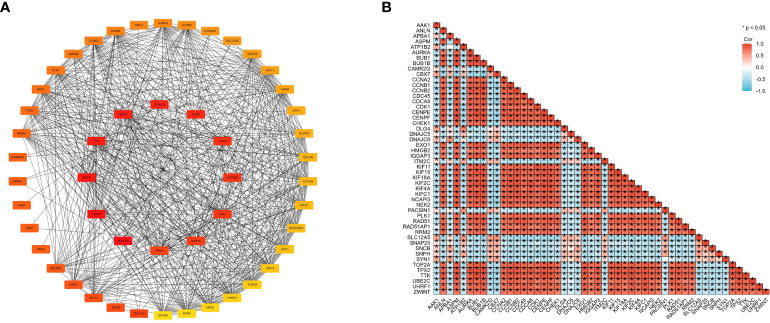
Cross-gene interactions were retrieved from the STRING database. The top 50 CENPE-related genes interaction network **(A)** and correlation matrix **(B)**.

We then used the R package tidyverse to further explore the biological function of CENPE. 5840 genes were positively correlated with the expression of CENPE, and 5272 genes were negatively correlated with the expression of CENPE (**Figure 4A**). Heat map showed the top 50 genes positively and negatively correlated with CENPE expression (**Figures 4B, C**). The top 200 co-expressed genes of CENPE were annotated by GO and KEGG functional enrichment analysis. The KEGG analysis showed that co-expression of CENPE was mainly involved Cell cycle, Human T-cell leukemia virus 1 infection, Cellular senescence, p53 signaling pathway, Fanconi anemia pathway (**Figure 4D**). GO functional annotations showed that CENPE co-expressed genes were mainly involved in the organelle fission (GO-BP) (**Figure 4E**), chromosomal region (GO-CC) (**Figure 4F**), tubulin binding (GO-MF) (**Figure 4G**). PPI network and correlated analysis were used to identify the interactions between the top 50 proteins related to CENPE. We found that most proteins in the network have strong positive or negative correlations with each other. (**Figures 5A, B**).

### Difference of biological pathways between high and low CENPE groups in non-WNT/non-SHH MB

**Figure 6:**
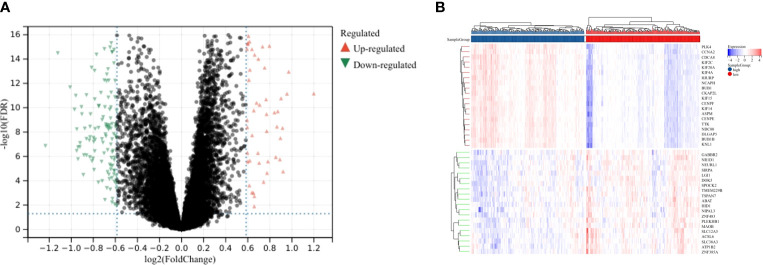
DEGs between CENPE^high^ and CENPE^low^ groups. **(A)** Volcano plot showing the DEGs according to the median expression of CENPE. **(B)** Heat map showing the top 20 upregulated and downregulated genes between CENPE^high^ and CENPE^low^ groups.

**Figure 7:**
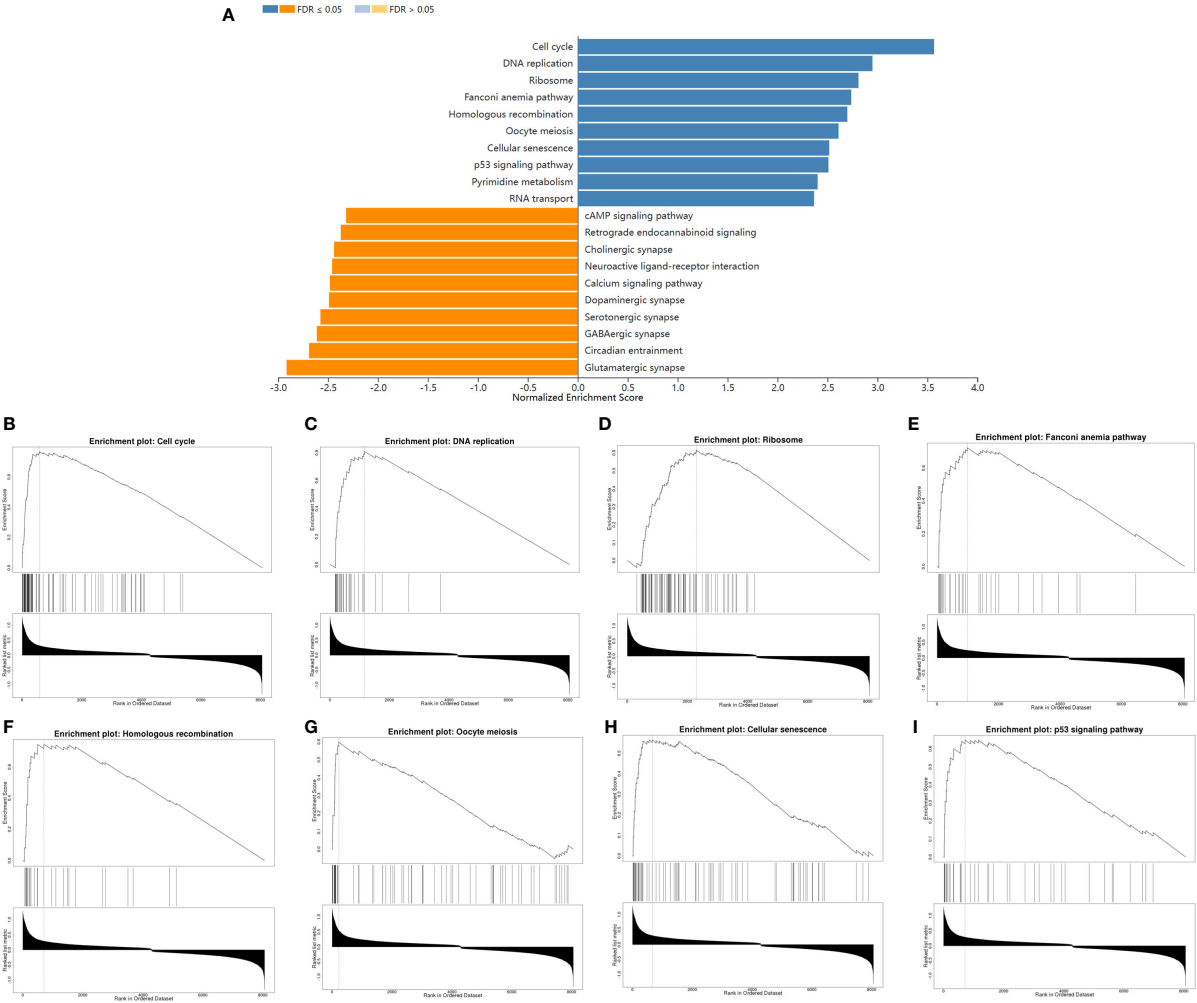
The differences in biological pathways between the CENPE^high^ and CENPE^low^ groups. **(A)** Bar graph showing the NES values of the top 10 KEGG pathways positively and negatively correlated with CENPE. **(B–I)** Enrichment plots showing the top 8 positively correlated KEGG pathways.

To further characterize the function of CENPE, we identified 8404 differentially expressed genes (DEGs) between the CENPE^high^ and CENPE^low^ non-WNT/non-SHH MB samples, including 4517 upregulated and 3887 downregulated genes (**Figure 6A**). The heat map showed the top 50 upregulated/downregulated genes (**Figure 6B**). Then the differentially expressed genes were used for KEGG analysis by GSEA. We ranked signal pathways based on standardized enrichment scores (**Figure 7A**). The eight KEGG-annotated pathways positively associated with high CENPE expression were in Cell cycle, DNA replication, Ribosome, Fanconi anemia pathway, Homologous recombination, Oocyte meiosis, Cellular senescence, p53 signaling pathway (**Figures 7B–I**). The above research results indicated that in non-WNT/non-SHH MB, the genes co expressed with CENPE and the DEGs between high and low CENPE groups both included cell cycle and P53 signaling pathway in their gene function enrichment results.

**Figure 8:**
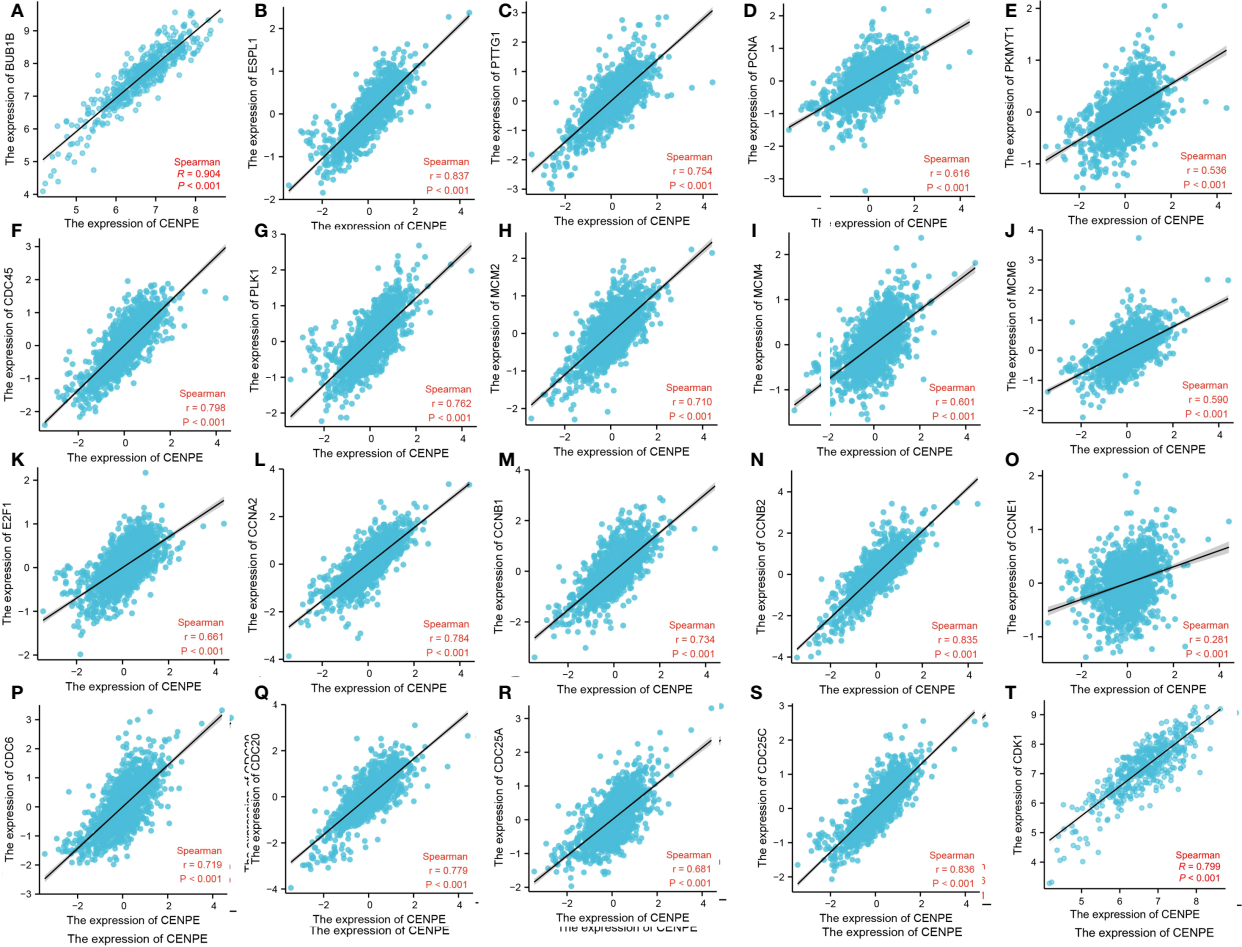
CENPE is closely related to cell cycle regulatory genes in non-WNT/non-SHH MB. **(A)** BUB1B, **(B)** ESPL1, **(C)** PTTG1, **(D)** PCNA, **(E)** PKMYT1, **(F)** CDC45, **(G)** PLK1, **(H)** MCM2, **(I)** MCM4, **(J)** MCM6, **(K)** E2F1, **(L)** CCNA2, **(M)** CCNB1, **(N)** CCNB2, **(O)** CCNE1, **(P)** CDC6, **(Q)** CDC20, **(R)** CDC25A, **(S)** CDC25C, **(T)** CDK1.

To further verify the relationship between CENPE and cell cycle, we analyzed the correlation between CENPE and cell cycle regulatory genes in non-WNT/non-SHH MB. The cycle regulatory genes BUB1B, ESPL1, PTTG1, PCNA, PKMYT1, CDC45, PLK1, MCM2, MCM4, MCM6, E2F1, CCNA2, CCNB1, CCNB2, CCNE1, CDC6, CDC20, CDC25A, CDC25C and CDK1 were positively correlated with CENPE (**Figure 8**).

### Knockdown of CENPE induced cell cycle arrest and p53 pathway activation in non-WNT/non-SHH MB

**Figure 9:**
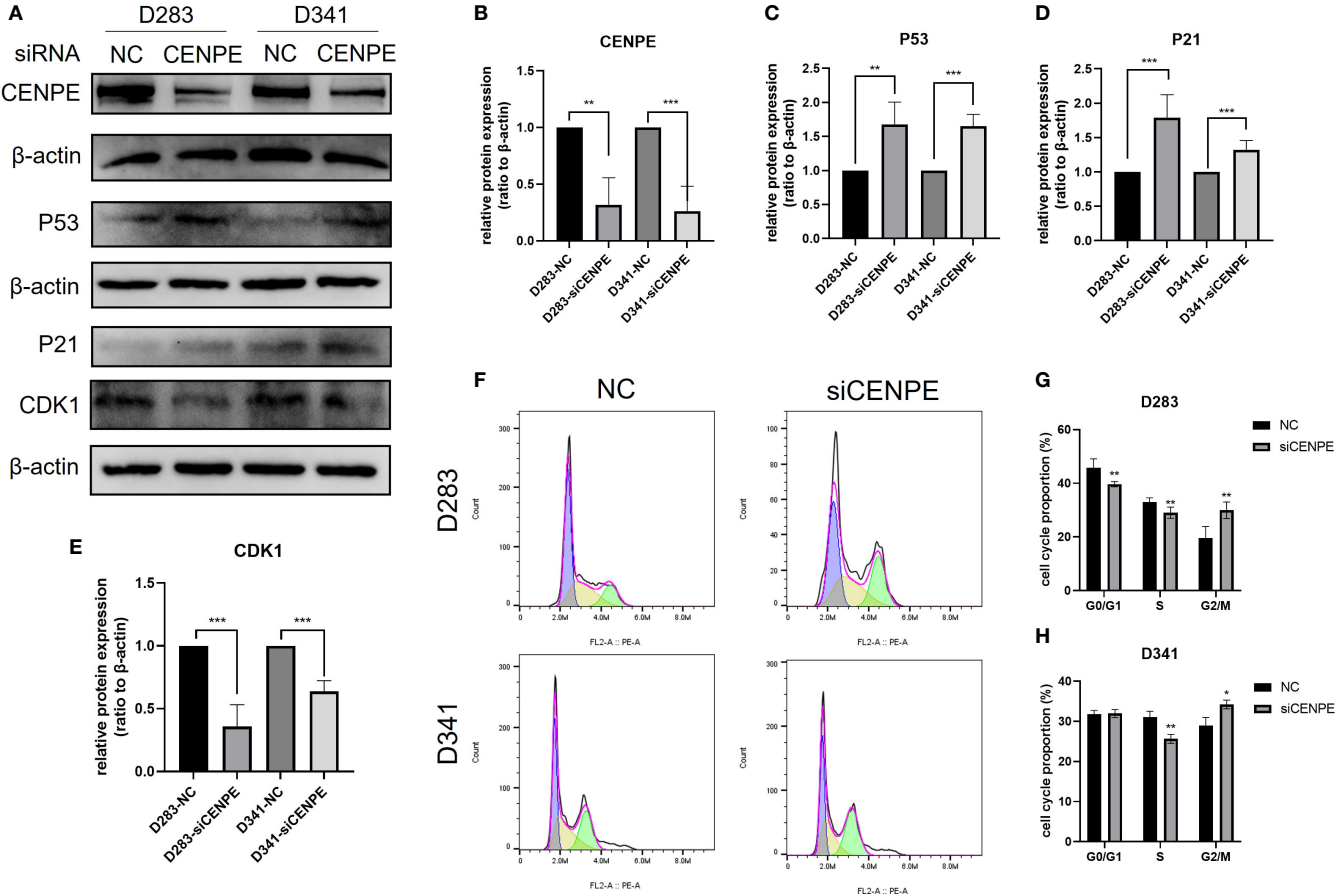
CENPE regulates the cell cycle and p53 pathway of non-WNT/non-SHH MB. **(A–E)** Western blot showed that the protein level of CENPE, P53, P21 and CDK1 by the knockdown of CENPE (mean ± SD, n = 3). **(F–H)** Flow cytometry was used to detect the cell cycle (mean ± SD, n = 3). *p < 0.05, **p < 0.01 and ***p < 0.001.

To explore the regulatory effect of CENPE on the cell cycle and p53 pathway, CENPE siRNA was used to interfere with CENPE expression. The protein expression levels of CENPE, p53, p21, and CDK1 were detected by Western blotting. The results showed that after transfection with CENPE, the expression of CENPE significantly decreased and the expression of p53 increased (**Figures 9A–C**). The cell cycle analysis revealed that knocking down CENPE caused accumulation of cells in G2/M phase in both non-WNT/non-SHH MB cell lines (**Figures 9F–H**). The expression of key factors p21 and CDK1 downstream of p53 responsible for cell cycle regulation (G2/M) had also changed. Compared with the control group, knocking down CENPE resulted in an increase in p21 expression (**Figures 9A, D**) and a decrease in CDK1 expression (**Figures 9A, E**). These data indicated that CENPE may regulate cell cycle through the p53 pathway.

### Knockdown of CENPE Inhibits the proliferation of non-WNT/non-SHH MB

**Figure 10:**
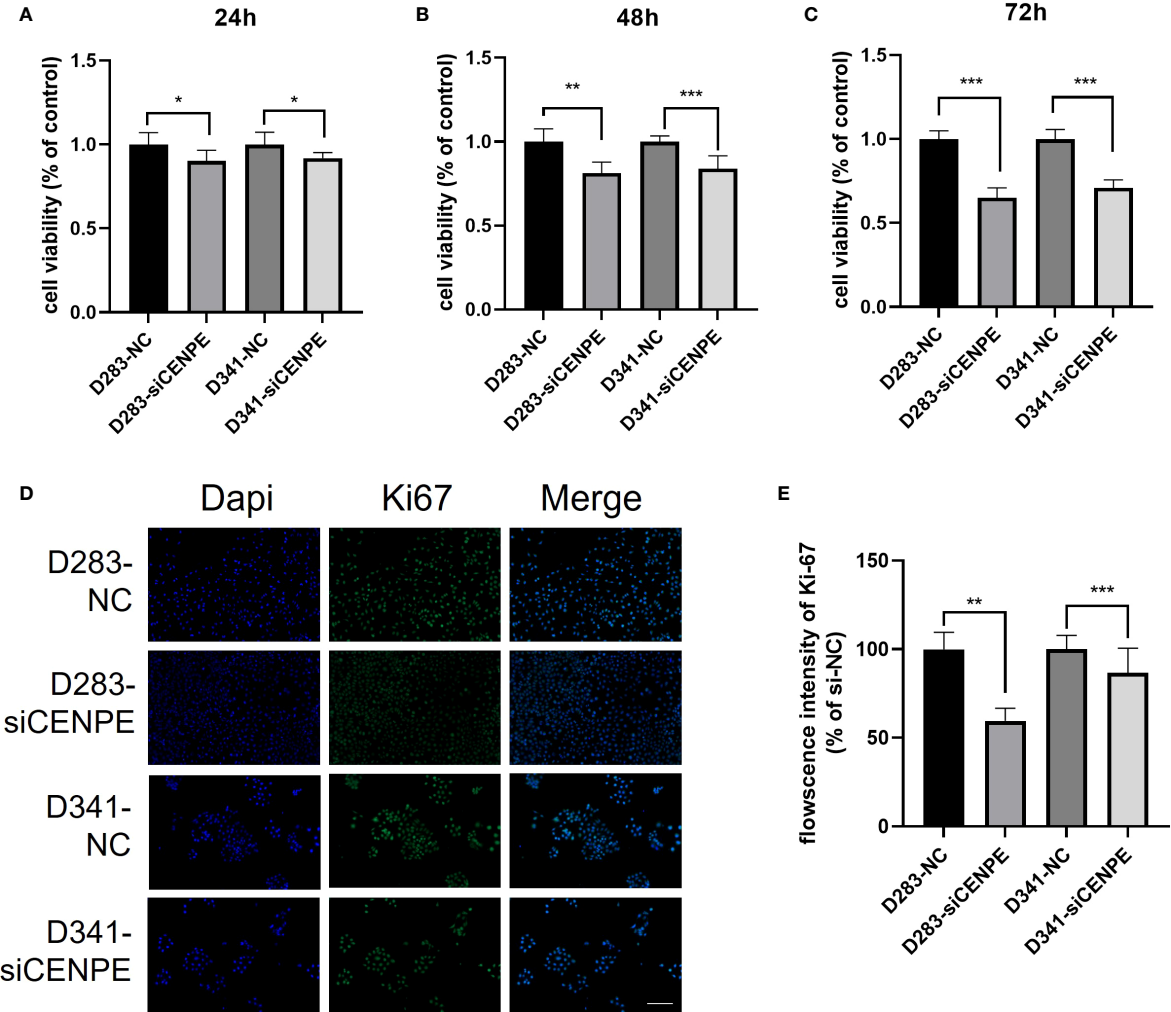
CENPE regulates the proliferation of non-WNT/non-SHH cells. **(A–C)** CCK-8 was used to evaluate the proliferation ability of D283 and D341 cells (mean ± SD, n = 3). **(D, E)** Immunofluorescence staining showed the Ki67 protein expression in D283 and D341 cells (scale bar, 100 μm), (mean ± SD, n = 3). *p < 0.05, **p < 0.01 and ***p < 0.001.

CCK-8 assays were performed to investigate the effect of CENPE on cell proliferation. The results showed that silencing CENPE significantly inhibited the proliferation of both cell lines at 24 h, 48 h, and 72 h after seeding (**Figures 10A–C**). In addition, immunofluorescence evaluation found that the average fluorescence intensity of cell proliferation protein Ki67 in the CENPE knockdown group was lower than that in the control group (**Figures 10D, E**). Based on the analysis of cell cycle, the inhibitory effect of knocking down CENPE on the proliferation of non-WNT/non-SHH MB cell lines may be partially mediated by cell cycle arrest.

## Discussion

Each subtype of MB has different molecular characteristics and targeted treatment strategies. A common strategy for developing new anticancer therapies is to directly target molecules that drive mutation changes. For example, various SHH pathway inhibitors, including Vimodji and Eremodeji, have been developed as a treatment for SHH subtype MB (17). Unfortunately, only some patients are sensitive to these drugs, and resistance may also occur in these patients (18, 19). In response to the high heterogeneity of non-WNT/non-SHH MB, another targeting strategy is represented by the class non-oncogene proteins that are essential for tumor growth and progression, despite not having mutations (20, 21). These protein molecules mediate biological changes during the process of cell carcinogenesis. In this study, we first reported that CENPE was overexpressed in MB, and CENPE had high sensitivity and specificity in distinguishing MB tissue from normal cerebellar tissue. In addition, our analysis suggests that CENPE was an independent risk factor for the prognosis of non-WNT/non-SHH MB patients. Patients with higher expression of CENPE had a shorter OS. These results indicate that CENPE is an important factor in the development of non-WNT/non-SHH MB.

As a microtubule plus-end-directed kinetochore protein, CENPE interacts with mitotic centromere-associated kinesin during mitosis to regulate chromosome-microtubule end-on attachment (6, 22). Microtubule is an important component of cytoskeleton, which is essential for cell division, vesicle transport and other cellular functions. CENPE heterozygous mutations can cause MCPH13 syndrome, characterized by reduced head circumference, sloping forehead and large ears (13, 23). In cancer treatments, various microtubule targeting agents (MTA), including paclitaxel and vincristine, have been developed for the treatment of cancer. However, due to the non-specific targeting of microtubules in cancer and normal cells by these drugs, the use of MTA in tumor treatment often leads to various adverse reactions such as neuropathy, bone marrow suppression, nausea, and febrile neutropenia-like septic death (21, 24, 25). Targeting protein molecules that act on microtubule dynamics but are only essential for cancer cells may be a specific target for MB therapy (26). They are necessary for the proliferation of neural progenitor cells and brain tumor cells. Inhibiting its function can mimic the effect of MTA without affecting the post mitotic cells of the central nervous system (27). This study analyzed the molecular mechanisms of tumor progression and poor prognosis mediated by CENPE in non-WNT/non-SHH MB. We conducted GO and KEGG functional enrichment analysis of CENPE co-expressed genes and found that the cell cycle gene set enrichment was most significant. This conclusion was also validated by differential genes GSEA analysis. Further analysis showed that CENPE was positively correlated with the expression of cell cycle regulator gene such as CCNB1 and CDK1. CDK1 plays an important role in the G2/M phase transition of cells (28). Our cell experiments showed that knockdown of CENPE can inhibit the expression of CDK1 and induce G2/M phase arrest in non-WNT/non-SHH MB cells.

In addition, pathway enrichment analysis also indicated a close correlation between CENPE and the p53 signaling pathway. As an important effector molecule downstream of the p53 pathway, p21 protein participates in the regulation of cell cycle (29). P21 inhibits cell G2/M phase transition by binding to the CCNB1/CDK1 complex to block the activation of cell division cycle factor 25 (CDC25) and cyclin dependent kinase activated kinases (CAK) (30). Our research results indicated that downregulation of CENPE can activate the p53 pathway and increase the expression levels of p53 and p21. The reason for P53 activation may be related to oblique cell division after loss of CENPE expression (31, 32). Therefore, we suggest that CENPE inhibition may exert a regulatory effect on cell cycle through the p53 pathway. The results of CCK-8 and Ki67 immunofluorescence staining indicated that CENPE affected the malignant proliferation of non-WNT/non-SHH MB cells by regulating the p53 pathway and cell cycle.

Tumor microenvironment (TME) plays an important role in the occurrence, development and metastasis of tumors (33). The regulatory effect of immune infiltrating cells in the tumor microenvironment on tumor cells can be a double-edged sword, which can inhibit tumor progress, and can also create an immunosuppressive microenvironment (34, 35). This study explored the correlation between CENPE expression and immune infiltration levels in non-WNT/non-SHH MB. The results showed that CENPE was positively correlated with the infiltration levels of aDC, B cells, CD4+ Tem, CD8+ Tcm, DC, conventional DC, immature DC, Macrophages, Macrophages M1, Mast cells, NK cells, pro B-cells, Tgd cells, Th1 cells and Th2 cells; while negatively correlated with the infiltration levels of CD4+ Tcm, Class-switched memory B cells, Eosinophils, NKT and Tregs. These findings suggest that CENPE may play an important role in the regulation of tumor immune microenvironment and serve as a potential immunotherapy related biomarker in non-WNT/non-SHH MB.

To summarize, our study found that the expression of CENPE was significantly upregulated and closely related to poor prognosis in patients with non-WNT/non-SHH MB. CENPE has reasonable accuracy in predicting the diagnosis and prognosis of non-WNT/non-SHH MB. CENPE may affect the progression of tumor by regulating cell cycle, p53 pathway, and immune infiltration. It can serve as a novel biomarker and potential therapeutic target for patients with non-WNT/non-SHH MB. However, this study still has some limitations. We lack direct evidence that CENPE affects prognosis through its involvement in immune infiltration, and the mechanism by which CENPE regulates immune infiltration needs to be elucidated through animal experiments.

## Data availability statement

The datasets presented in this study can be found in online repositories. The names of the repository/repositories and accession number(s) can be found in the article/supplementary material.

## Author contributions

HF, JL, and HS conceived the study. HF, YZ, CL, JL, and HS designed the study. HF, YZ, CL, ZS, and WW performed experiments. HF, YZ, and CL analyzed the data. HF and YZ drafted the manuscript. HF, CL, and JL revised the manuscript. All authors contributed to the article and approved the submitted version.

## References

[1] Ostrom Q Patil N Cioffi G Waite K Kruchko C Barnholtz-Sloan J. CBTRUS statistical report: primary brain and other central nervous system tumors diagnosed in the United States in 2013-2017. Neuro-oncology (2020) 22:iv1–iv96. doi: 10.1093/neuonc/noaa200 33123732 PMC7596247

[2] Taylor M Northcott P Korshunov A Remke M Cho Y Clifford S. Molecular subgroups of medulloblastoma: the current consensus. Acta neuropathol (2012) 123(4):465–72. doi: 10.1007/s00401-011-0922-z PMC3306779 22134537

[3] Louis D Perry A Wesseling P Brat D Cree I Figarella-Branger D. The 2021 WHO classification of tumors of the central nervous system: a summary. Neuro-oncology (2021) 23(8):1231–51. doi: 10.1093/neuonc/noab106 PMC8328013 34185076

[4] Northcott P Jones D Kool M Robinson G Gilbertson R Cho Y. Medulloblastomics: the end of the beginning. Nat Rev Cancer (2012) 12(12):818–34. doi: 10.1038/nrc3410 PMC3889646 23175120

[5] Cooney T Lindsay H Leary S Wechsler-Reya R. Current studies and future directions for medulloblastoma: A review from the pacific pediatric neuro-oncology consortium (PNOC) disease working group. Neoplasia (New York NY). (2023) 35:100861. doi: 10.1016/j.neo.2022.100861 PMC9755363 36516489

[6] Yu K Zhong N Xiao Y She Z. Mechanisms of kinesin-7 CENP-E in kinetochore-microtubule capture and chromosome alignment during cell division. Biol Cell (2019) 111(6):143–60. doi: 10.1111/boc.201800082 30784092

[7] Yen T Compton D Wise D Zinkowski R Brinkley B Earnshaw W. CENP-E, a novel human centromere-associated protein required for progression from metaphase to anaphase. EMBO J (1991) 10(5):1245–54. doi: 10.1002/j.1460-2075.1991.tb08066.x PMC452779 2022189

[8] Fang L Liu Q Cui H Zheng Y Wu C. Bioinformatics analysis highlight differentially expressed CCNB1 and PLK1 genes as potential anti-breast cancer drug targets and prognostic markers. Genes (2022) 13(4):654. doi: 10.3390/genes13040654 35456460 PMC9027215

[9] Hao X Qu T. Expression of CENPE and its prognostic role in non-small cell lung cancer. Open Med (Warsaw Poland) (2019) 14:497–502. doi: 10.1515/med-2019-0053 PMC6592151 31259255

[10] Zhang Z Zhang X. Identification of m6A-related biomarkers associated with prognosis of colorectal cancer. Med Sci monitor (2021) 27:e932370. doi: 10.12659/MSM.932370 PMC8364289 34373442

[11] Shan L Zhao M Lu Y Ning H Yang S Song Y. CENPE promotes lung adenocarcinoma proliferation and is directly regulated by FOXM1. Int J Oncol (2019) 55(1):257–66. doi: 10.3892/ijo.2019.4805 31115500

[12] Li J Diao H Guan X Tian X. Kinesin family member C1 (KIFC1) regulated by centrosome protein E (CENPE) promotes proliferation, migration, and epithelial-mesenchymal transition of ovarian cancer. Med Sci monitor (2020) 26:e927869. doi: 10.12659/MSM.927869 PMC7780892 33361741

[13] Mirzaa G Vitre B Carpenter G Abramowicz I Gleeson J Paciorkowski A. Mutations in CENPE define a novel kinetochore-centromeric mechanism for microcephalic primordial dwarfism. Hum Genet (2014) 133(8):1023–39. doi: 10.1007/s00439-014-1443-3 PMC4415612 24748105

[14] Iegiani G Gai M Di Cunto F Pallavicini G. CENPE inhibition leads to mitotic catastrophe and DNA damage in medulloblastoma cells. Cancers (2021) 13(5):1028. doi: 10.3390/cancers13051028 33804489 PMC7957796

[15] Gibson P Tong Y Robinson G Thompson M Currle D Eden C. Subtypes of medulloblastoma have distinct developmental origins. Nature (2010) 468(7327):1095–9. doi: 10.1038/nature09587 PMC3059767 21150899

[16] Ivanov D Coyle B Walker D Grabowska A. *In vitro* models of medulloblastoma: Choosing the right tool for the job. J Biotechnol (2016) 236:10–25. doi: 10.1016/j.jbiotec.2016.07.028 27498314

[17] Samkari A White J Packer R. SHH inhibitors for the treatment of medulloblastoma. Expert Rev Neurother (2015) 15(7):763–70. doi: 10.1586/14737175.2015.1052796 26027634

[18] Robinson G Orr B Wu G Gururangan S Lin T Qaddoumi I. Vismodegib exerts targeted efficacy against recurrent sonic hedgehog-subgroup medulloblastoma: results from phase II pediatric brain tumor consortium studies PBTC-025B and PBTC-032. J Clin Oncol (2015) 33(24):2646–54. doi: 10.1200/JCO.2014.60.1591 PMC4534527 26169613

[19] Thompson E Ashley D Landi D. Current medulloblastoma subgroup specific clinical trials. Trans pediatrics (2020) 9(2):157–62. doi: 10.21037/tp.2020.03.03 PMC7237968 32477916

[20] Luo J Solimini N Elledge S. Principles of cancer therapy: oncogene and non-oncogene addiction. Cell (2009) 136(5):823–37. doi: 10.1016/j.cell.2009.02.024 PMC2894612 19269363

[21] Iegiani G Di Cunto F Pallavicini G. Inhibiting microcephaly genes as alternative to microtubule targeting agents to treat brain tumors. Cell Death Dis (2021) 12(11):956. doi: 10.1038/s41419-021-04259-6 34663805 PMC8523548

[22] Wood K Sakowicz R Goldstein L Cleveland D. CENP-E is a plus end-directed kinetochore motor required for metaphase chromosome alignment. Cell (1997) 91(3):357–66. doi: 10.1016/S0092-8674(00)80419-5 9363944

[23] Fagerberg L Hallström B Oksvold P Kampf C Djureinovic D Odeberg J. Analysis of the human tissue-specific expression by genome-wide integration of transcriptomics and antibody-based proteomics. Mol Cell proteomics: MCP (2014) 13(2):397–406. doi: 10.1074/mcp.M113.035600 24309898 PMC3916642

[24] Mukhtar E Adhami V Mukhtar H. Targeting microtubules by natural agents for cancer therapy. Mol Cancer Ther (2014) 13(2):275–84. doi: 10.1158/1535-7163.MCT-13-0791 PMC3946048 24435445

[25] Gornstein E Schwarz T. The paradox of paclitaxel neurotoxicity: Mechanisms and unanswered questions. Neuropharmacology (2014), 175–83. doi: 10.1016/j.neuropharm.2013.08.016 23978385

[26] Lang P Gershon T. A New Way to Treat Brain Tumors: Targeting Proteins Coded by Microcephaly Genes?: Brain tumors and microcephaly arise from opposing derangements regulating progenitor growth. Drivers of microcephaly could be attractive brain tumor targets. BioEssays (2018) 40(5):e1700243. doi: 10.1002/bies.201700243 29577351 PMC5910257

[27] O'Neill R Schoborg T Rusan N. Same but different: pleiotropy in centrosome-related microcephaly. Mol Biol Cell (2018) 29(3):241–6. doi: 10.1091/mbc.E17-03-0192 PMC5996963 29382806

[28] Ruiz-Losada M González R Peropadre A Gil-Gálvez A Tena J Baonza A. Coordination between cell proliferation and apoptosis after DNA damage in Drosophila. Cell Death Differ (2022) 29(4):832–45. doi: 10.1038/s41418-021-00898-6 PMC8989919 34824391

[29] Engeland K. Cell cycle regulation: p53-p21-RB signaling. Cell Death Differ (2022) 29(5):946–60. doi: 10.1038/s41418-022-00988-z PMC9090780 35361964

[30] Choi W Kim M Jeon B Koh D Yun C Li Y. Role of promyelocytic leukemia zinc finger (PLZF) in cell proliferation and cyclin-dependent kinase inhibitor 1A (p21WAF/CDKN1A) gene repression. J Biol Chem (2014) 289(27):18625–40. doi: 10.1074/jbc.M113.538751 PMC4081908 24821727

[31] Owa M Dynlacht B. A non-canonical function for Centromere-associated protein-E controls centrosome integrity and orientation of cell division. Commun Biol (2021) 4(1):358. doi: 10.1038/s42003-021-01861-4 33742057 PMC7979751

[32] Yu K She Z Wei Y Zhong N. Kinesin-7 CENP-E regulates cell division, gastrulation and organogenesis in development. Eur J Cell Biol (2020) 99(6):151107. doi: 10.1016/j.ejcb.2020.151107 32800279

[33] Wang M Chang M Li C Chen Q Hou Z Xing B. Tumor-microenvironment-activated reactive oxygen species amplifier for enzymatic cascade cancer starvation/chemodynamic /immunotherapy. Adv Mater (Deerfield Beach Fla) (2022) 34(4):e2106010. doi: 10.1002/adma.202106010 34699627

[34] Bruno T. New predictors for immunotherapy responses sharpen our view of the tumour microenvironment. Nature (2020) 577(7791):474–6. doi: 10.1038/d41586-019-03943-0 PMC7523515 31965091

[35] Jiang X Wang J Deng X Xiong F Ge J Xiang B. Role of the tumor microenvironment in PD-L1/PD-1-mediated tumor immune escape. Mol cancer (2019) 18(1):10. doi: 10.1186/s12943-018-0928-4 30646912 PMC6332843

